# Strongyloides Infection Presenting as *E. coli* Meningitis Post-Transplant

**DOI:** 10.3390/tropicalmed10050143

**Published:** 2025-05-20

**Authors:** Anusha Belani, Chaitanya Mishra, Daniela Mihova, Todd Gleeson, Anita Nahar

**Affiliations:** 1 Department of Medicine, Frederick Health Hospital, 400 W 7th Street, Frederick, MD 21701, USA; 2 Walter Reed National Military Medical Center, 4494 Palmer Rd N, Bethesda, MD 20814, USA

**Keywords:** strongyloidiasis, meningitis, transplantation

## Abstract

Strongyloidiasis is a common intestinal infection that may persist in humans for decades without symptoms or can present as a potentially fatal, broadly disseminated infection in an immunocompromised host. This report describes a rare case of concomitant strongyloidiasis and *E. coli* meningitis that was successfully recognized and treated in a patient 20 years post-renal transplantation.

## 1. Introduction

Strongyloidiasis is a parasitic infection caused by *Strongyloides stercoralis*, which affects over 100 million people globally [1,2]. Risk factors for exposure include travel to endemic areas, including Southeast Asia, where countries have reported an average 30% seroprevalence of *S. stercoralis*. The rural highlands of Vietnam have the highest rate in the region of 42% [3]. Within the United States, the Southeast has been linked to strongyloidiasis from exposure to contaminated soil (e.g., walking barefoot, gardening without gloves) [4]. In addition, dogs and humans share certain *S. stercoralis* genotypes, and there are case reports supporting the hypothesis of transmission from dogs (reservoir) to humans [5,6]. However, the zoonotic potential between these two hosts is controversial and not well understood [7]. The estimated global prevalence of *S. stercoralis* in dogs is 6%, with prevalence in lower-income countries higher at 22% [5].

This parasite is seldom regarded as a primary contributor to meningitis in patients who have undergone renal transplantation. Additionally, organ system involvement and symptoms of strongyloidiasis can vary. A transplanted host on active immunosuppression may provide an easy migration path for the parasite from the skin and gastrointestinal tract to the lungs and meninges [8]. Symptoms can be indolent for years and can be non-specific, including abdominal pain, diarrhea, constipation, weight loss, nausea, and gastrointestinal bleeding [9]. Pulmonary symptoms may include cough, wheezing, chest pain, and hemoptysis. Meningeal inflammation as a result of direct invasion of *S. stercoralis* can mimic aseptic or bacterial meningitis [10], and concomitant bacteremia and meningeal inflammation are seen in 50% of cases [11].

Here, we aim to highlight the importance of considering strongyloidiasis as a risk factor for Gram-negative meningitis. This report is in alignment with NEJM HIPAA policies, and consent was obtained from the patient for publication of this case.

## 2. Case Presentation

Our patient was a 77-year-old (83.6 kg) Caucasian male with a history of hypertension and end-stage renal disease (ESRD) secondary to congenital atrophy (left) and traumatic kidney injury (right) from a motor vehicle accident, now status-post living-related kidney transplant (20 years prior). He was maintained on immunosuppressive therapy with tacrolimus, mycophenolate, and steroids, with a stable creatinine of 1.2 mg/dL. Additional history included a cochlear implant and history of a neurogenic bladder from lumbar disk disease, requiring self-catheterization and trimethoprim for urinary tract infection (UTI) prophylaxis.

The patient initially presented with a fever and delusions, and he quickly became encephalopathic and hypoxic, necessitating urgent intubation. His highest temperature was recorded at 103.9 °F, raising concerns for acute meningitis. A diagnostic lumbar puncture was performed, and results were suspicious for a suppurative central nervous system (CNS) infection, as the cerebrospinal fluid (CSF) obtained was yellow and cloudy, with an opening pressure of 37 mm H_2_O. Initial bloodwork revealed a white blood cell (WBC) count of 17.5 × 10^9^/L with 90% neutrophils, a blood urea nitrogen (BUN) level of 39 mg/dL, and a creatinine level of 1.5 mg/dL. Eosinophil count was 0 cells/mcL (0%) in the setting of chronic immunosuppressive therapy. Procalcitonin level was 54.6 ng/mL, and Coronavirus disease (COVID-19) and influenza (A and B) polymerase chain reaction (PCR) testing were negative. Dexamethasone, cefepime, ampicillin, vancomycin, and acyclovir were initiated empirically for broad coverage. Mycophenolate was discontinued on day 1, while tacrolimus was stopped on day 8.

Findings from the initial CSF in tube #3 are shown in Table 1 (first column). Blood, urine, and spinal fluid cultures all grew out pan-sensitive *Escherichia coli* on follow-up, concerning for an *E. coli* UTI and septicemia with meningeal seeding. A computed tomography (CT) scan of the chest, abdomen, and pelvis with intravenous contrast showed the transplanted kidney in the right lower quadrant without hydronephrosis, and non-specific bladder wall thickening without other acute evidence of an ascending UTI. In addition, there was no clinical or radiological evidence of an epidural abscess or discitis.

A transthoracic echocardiogram showed an ejection fraction of 65–70%, no significant valvular lesions, and no obvious vegetations. His high-dose steroid was discontinued after 2 days. Due to concerns for seeding of his cochlear implant, a repeat lumbar puncture was performed after 48 h of Gram-negative coverage to assess efficacy. His spinal fluid WBC count remained elevated at 2150, with 98% leukocytes, a glucose level of 21 mg/100 mL, and a CSF protein level of 347 mg/100 mL. CSF cultures were persistently positive for *E. coli*. Cefepime was changed to ceftriaxone based on blood culture susceptibilities on hospital day #4. The CSF continued to have similar characteristics and remained *E. coli* positive on a repeat lumbar puncture conducted 5 days after initiation of Gram-negative treatment, and levofloxacin was added the same day for dual Gram-negative coverage.

**Figure 1:**
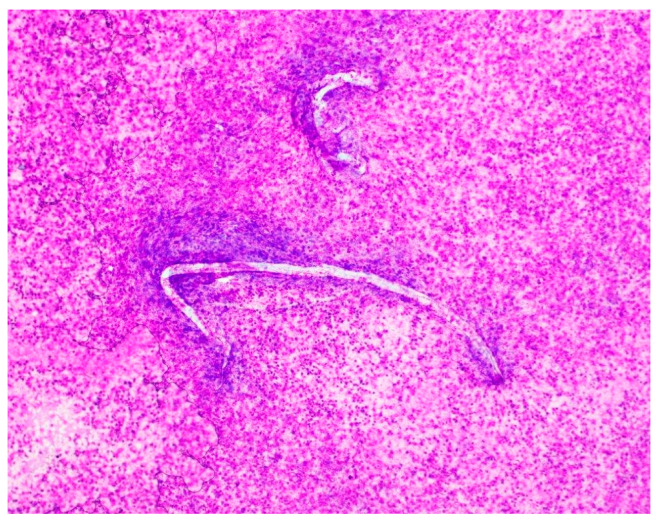
Hospital day #1, Magnification 20×; Diff-Quik stain. Cerebrospinal fluid (CSF) shows filariform larvae of *Strongyloides stercoralis* observed by microscopy of the cerebrospinal fluid from the patient. In the background, there is a massive load of rod-shaped *E. coli* organisms and numerous acute inflammatory cells.

The patient remained intubated. Given his unresolved clinical picture, the patient’s wife was re-interviewed, at which point she revealed significant additional history. The patient had traveled to Vietnam 6 years prior, had been on several cruises in the past 6 years (West Caribbean, Mexico, Hawaii), and routinely traveled to Florida each summer. She also recounted that they had a 7-year-old pet dog that had vomited on the carpet prior to the patient’s illness, and he had cleaned the carpet after this event. Their dog was taken to the veterinarian and later passed away from unspecified multisystem organ failure. In light of this new information, the hospital pathologist was asked to review the CSF slides for *Strongyloides*. At this time, filariform larvae were noted on all slides (Figure 1). A heavy burden of inflammatory cells and bacteria was present despite 6 days of antibiotics.

Serum *Strongyloides* antibody titer was positive on hospital day #6 at 0.5 IV (reference < 0.1 IV), and the patient was started on ivermectin 18 mg/day (200 mcg/kg body weight) via nasogastric tube. Additional *Strongyloides* testing was attempted from tracheal secretions, gastric contents, and duodenal biopsies, which all returned negative. The stool Strongyloidiasis PCR was not available at our facility or reference lab. Further CSF testing indicated that the CSF was negative for cytomegalovirus (CMV), *Cryptococcus*, *Histoplasma*, Herpes simplex virus (HSV), and human T-cell lymphotropic virus types 1 and 2 (HTLV-1 and HTLV-2). CSF immunoglobulin panels were also normal.

Ivermectin, ceftriaxone, and levofloxacin were continued, with serial lumbar punctures conducted every 3–4 days. The patient stabilized and was extubated on hospital day #9. CSF cell counts decreased on serial studies, but persistent evidence of *Strongyloides* larvae and filamentous structures was noted. On hospital day #10, albendazole 400 mg twice a day was initiated for a 7-day course. In light of continued pleocytosis despite adequate therapy, a full workup for autoimmune conditions, *Tropheryma whipplei,* and BioFire film array was initiated, but all returned negative.

The patient received a total of 5 weeks of ivermectin, given his initial heavy load of *Strongyloides* and continued pleocytosis in addition to 6 weeks of coverage for *E. coli* meningitis. His immunosuppression was held for the entire duration, and renal function remained normal. He was resumed on tacrolimus 0.5 mg once a day before discharge, and was discharged home after a 48-day admission. Overall, he demonstrated no signs of neurotoxicity from prolonged ivermectin therapy. At the 6-week outpatient mark, his tacrolimus dosage was increased to 0.5 mg twice daily, and mycophenolate was permanently discontinued due to concerns about the potential recurrence of strongyloidiasis.

## 3. Discussion

Our renal transplant patient presented with late strongyloidiasis superimposed on *E. coli* bacteremia and meningitis, requiring prolonged therapy with Ivermectin in addition to routine therapies indicated for Gram-negative bacteremia. Subsequently, we learned that his non-specific symptoms (pulmonary infections and diarrheal episodes) were ongoing for more than 9 months prior to presentation. CNS involvement later during the described admission was what prompted further workup.

A manifestation of strongyloidiasis as Gram-negative meningitis is rare and the proposed mechanism is multi-faceted, involving disruption of the gastrointestinal mucosa by the parasite, followed by direct entry of bowel flora into the bloodstream and subsequently translocation of bowel flora with the infected larvae across the blood–brain barrier after repeated bacteremic episodes [12,13].

His risk factors included travel to endemic areas, an immunosuppressed status, and a possible exposure via his pet dog. As disseminated strongyloidiasis carries a mortality rate of almost 80%, early diagnosis and treatment were imperative to maximize chances of recovery. There is currently no gold standard test for making this diagnosis.

This case highlights a need to formalize screening and testing for re-infection in transplant patients. Organ procurement organizations are not currently required to test donors for *Strongyloides* [14], although transplant organizations have made an effort since 2010 to extend practical recommendations for potential recipient screening [15,16]. These algorithms, in addition to recommendations from the Centers for Disease Control and Prevention (CDC), Infectious Diseases Society of America (IDSA), and American Society of Transplantation (AST) include suggestions for targeted donor and recipient screening based on travel to or residence in an endemic area, demographic risk factors, animal exposures, or evidence of peripheral eosinophilia (present in approximately 20% of *Strongyloides*-seropositive patients [17]). Screening should be conducted with serological testing by enzyme-linked immunosorbent assay (ELISA) or with stool examination. ELISA testing has 84–95% sensitivity and 82–100% specificity [18,19]. Of significant note, post-transplant monitoring guidelines for *Strongyloides* currently do not exist. We suggest post-transplant screening of recipients with a detailed questionnaire, including travel, exposures, and recreational activities, in order to prevent future hyperinfection. Testing should also be quickly performed when suspicion is high for *Strongyloides*. Among the available tests (stool exams, molecular diagnostics, and serological tests), the droplet digital polymerase chain reaction (ddPCR) assay to detect *S. stercoralis* larvae may provide the best sensitivity and specificity for detection in stool samples [20], although this testing is not widely available. Use of corticosteroid therapy in this patient population may mask blunt high peripheral eosinophil counts, and as such, this particular marker should be used with caution in marker for screening.

The choice of drug therapy in our patient was carefully selected, given the circumstances of the late presentation. In general, treatment options for strongyloidiasis include albendazole, thiabendazole, and ivermectin [21]. Overall, ivermectin is well tolerated, with minimal side effects and an association with greater eradication of parasite larvae when compared to albendazole. Because of continued evidence of parasites in the CNS, we chose to use it in combination with albendazole. There is limited data on the duration of treatment for CNS strongyloidiasis and treatment of hyperinfection in immunocompromised patients. The CDC recommends ivermectin 200 ug/kg per day orally, until stool and/or sputum exams are negative for 2 weeks, along with suspension of immunosuppressive therapy. A combination of ivermectin and albendazole 10 mg/kg/day has been utilized in case reports and is reasonable given the severity of illness, despite the lack of high-quality data comparing this approach to ivermectin monotherapy [22,23]. Maintenance after initial therapy has been described with 2 doses of ivermectin at 200 mg/kg every 2 weeks for 6 weeks in immunocompromised patients to ensure microscopic clearance of larvae from infected sites [24], prevent recurrence of hyperinfection, and achieve adequate future prophylaxis [25]. Given the severity of illness in these patients and in the setting of suboptimal gastrointestinal absorption, administration of subcutaneous ivermectin can also be considered.

Duration of therapy was guided by our patient’s prolonged pleocytosis attributed to *Strongyloides* presence in the CSF. We opted for a longer course of Ivermectin (total duration of 5 weeks; until CSF clearance + an additional 14 days), in order to assure elimination of the larvae and prevent recurrence, as a development cycle takes 2 weeks [26]. Ivermectin was tolerated well in our patient without toxicity or side effects. Although CSF cultures returned negative for bacteria on hospital day #24, filariform organisms were present on slides until day #35. Of note, the patient received an initial short course of high-dose corticosteroids for meningitis and respiratory failure, and it remains unclear whether this short steroid burst played a role in promoting the transition of *S. stercolaris* larvae from the rhabdiform stage to the filariform stage, thus delaying the clearance of infection.

## 4. Conclusions

We demonstrate here a case of late disseminated strongyloidiasis post-transplant, with an initial presentation of *E. coli* urosepsis and acute meningitis. Disseminated strongyloidiasis is fatal in immunocompromised hosts, but initiation of dual drug therapy allowed our particular patient to have a great outcome.

For early diagnosis, we suggest screening with history including travel, recreational activities, and high steroid use, and early labs looking for eosinophilia. The protocols for screening in this patient population need to be adjusted.

## Figures and Tables

**Table 1:** Patient CSF findings by hospital day.

CSF Characteristic, with Normal Values/Ranges	Hospital Day 1 (Admission)	Hospital Day 4	Day 7	Day 10	Day 21	Day 28	Day 35	Day 45
Tube number	3	3	3	1	3	4	3	2
Color	Yellow	Yellow	Yellow	Colorless	Colorless	Colorless	Colorless	Colorless
RBC (0–5/cmm)	507	296	22	1200	130	0	215	44
WBC (0–5/cmm)	2070	2150	819	6	169	26	202	60
Polymorphonuclear (0–7%)	88	98	86	93	56	94	77	8
Lymphocytes (28–96%)	1		10	6	24	5	22	85
Monos/Macrophages (16–56%)	11	2	4	1	20	1	1	7
Glucose (40–70 mg/dL)	<2	21	40	30	137	104	74	60
Total Protein (15–45 mg/dL)	>600	347	550	>600	109	136	99	78

## Data Availability

No new data were created or analyzed in this study. Data sharing is not applicable to this article.

## References

[1] Genta R.M. (1989). Global prevalence of strongyloidiasis: Critical review with epidemiologic insights into the prevention of disseminated disease. Rev. Infect. Dis..

[2] Puthiyakannon S., Boddu A., Li Y., Zhou X., Wang C., Li J., Chen X. (2014). Strongyloidiasis--an insight into its global prevalence and management. PLoS Negl. Trop. Dis..

[3] Diep N.T.N., Thai P.Q., Trang N.N.M., Jäger J., Fox A., Horby P., Phuong H.V.M., Anh D.D., Mai L.T.Q., Doorn H.R.V. (2017). trongyloides stercoralis seroprevalence in Vietnam. Epidemiol. Infect..

[4] Centers for Disease Control and Prevention Parasites-Strongyloides. Epidemiology and Risk Factors. https://www.cdc.gov/strongyloides/about/?CDC_AAref_Val=https://www.cdc.gov/parasites/strongyloides#cdc_disease_basics_risk-risk-factors.

[5] Eslahi A.V., Hashemipour S., Olfatifar M., Houshmand E., Hajialilo E., Mahmoudi R., Badri M., Ketzis J.K. (2022). Global prevalence and epidemiology of Strongyloides stercoralis in dogs: A systematic review and meta-analysis. Parasites Vectors.

[6] Barratt J.L.N., Lane M., Talundzic E., Richins T., Robertson G., Formenti F., Pritt B., Verocai G., de Souza J.N., Soares N.M. (2019). A global genotyping survey of Strongyloides stercoralis and Strongyloides fuelleborni using deep amplicon sequencing. PLoS Negl. Trop. Dis..

[7] Jaleta T.F., Zhou S., Bemm F.M., Schär F., Khieu V., Muth S., Odermatt P., Lok J.B., Streit A. (2017). Strongyloides stercoralis in dogs and humans-Dogs as a possible source for zoonotic strongyloidiasis. PLoS Negl. Trop. Dis..

[8] Dutcher J.P., Marcus S.L., Tanowitz H.B., Wittner M., Fuks J.Z., Wiernik P.H. (1990). Disseminated strongyloidiasis with central nervous system involvement diagnosed antemortem in a patient with acquired immunodeficiency syndrome and Burkitts lymphoma. Cancer.

[9] Keiser P.B., Nutman T.B. (2004). Strongyloides stercoralis in the Immunocompromised Population. Clin. Microbiol. Rev..

[10] Vishwanath S., Baker R.A., Mansheim B.J. (1982). Strongyloides infection and meningitis in an immunocompromised host. Am. J. Trop. Med. Hyg..

[11] Hayes J., Nellore A. (2018). Management of Strongyloides in Solid Organ Transplant Recipients. Infect. Dis. Clin. N. Am..

[12] Link K., Orenstein R. (1999). Bacterial complications of strongyloidiasis: Streptococcus bovis meningitis. South. Med. J..

[13] Shorman M., Al-Tawfiq J.A. (2009). Strongyloides stercoralis hyperinfection presenting as acute respiratory failure and Gram-negative sepsis in a patient with astrocytoma. Int. J. Infect. Dis..

[14] Abad C.K.R., Bhaimia E., Schueta A.N., Razonable R.R. (2022). A comprehensive review of Strongyloides stercoralis infection after solid organ and hematopoietic stem cell transplantation. Clin. Transplant..

[15] Clemente W.T., Pierrotti L.C., Abdala E., Morris M.I., Azevedo L.S., López-Vélez R., Cuenca-Estrella M., Torre-Cisneros J., Petersen E., Camargo L.F.A. (2018). Recommendations for Management of Endemic Diseases and Travel Medicine in Solid-Organ Transplant Recipients and Donors: Latin America. Transplantation.

[16] Fischer S.A., Lu K. (2013). Screening of donor and recipient in solid organ transplantation. Am. J. Transplant..

[17] Loutfy M.P., Wilson M., Keystone J.S., Kain K.C. (2002). Serology and eosinophil count in the diagnosis and management of strongyloidiasis in a non-endemic area. Am. J. Trop. Med. Hyg..

[18] Malinis M., Boucher H.W. (2019). Screening of donor and candidate prior to solid organ transplantation-Guidelines from the American Society of Transplantation Infectious Diseases Community of Practice. Clin. Transplant..

[19] La Hoz Morris M. (2019). Intestinal parasites including Cryptosporidium, Cyclospora, Giardia, and Microsporidia, Entamoeba histolytica, Strongyloides, Schistosomiasis, and Echinococcus: Guidelines from the American Society of Transplantation Infectious Diseases Community of Practice. Clin. Transplant..

[20] Iamrod K., Chaidee A., Rucksaken R., Kopolrat K.Y., Worasith C., Wongphutorn P., Intuyod K., Pinlaor S., Sithithaworn J., Sithithaworn P. (2021). Development and Efficacy of Droplet Digital PCR for Detection of Strongyloides stercoralis in Stool. Am. J. Trop. Med. Hyg..

[21] Henriquez-Camacho C., Gotuzzo E., Echevarria J., White Jr A.C., Terashima A., Samalvides F., Pérez-Molina J.A., Plana M.N. (2016). Ivermectin versus albendazole or thiabendazole for Strongyloides stercoralis infection. Cochrane Database Syst. Rev..

[22] Mejia R., Nutman T.B. (2012). Screening, prevention, and treatment for hyperinfection syndrome and disseminated infections caused by Strongyloides stercoralis. Curr. Opin. Infect. Dis..

[23] Pornsuriyasak P., Niticharoenpong K., Sakapibunnan A. (2004). Disseminated strongyloidiasis successfully treated with extended duration ivermectin combined with albendazole: A case report of intractable strongyloidiasis. Southeast Asian J. Trop. Med. Public Health.

[24] Pedersen A.A., Hartmeyer G.N., Stensvold C.R., Martin-Iguacel R. (2022). *Strongyloides stercoralis* hyperinfection syndrome with cerebral involvement. BMJ Case Rep..

[25] Mirdha B.R. (2009). Human strongyloidiasis: Often brushed under the carpet. Trop. Gastroenterol..

[26] Miller M.A., Churhc L.W.P., Salgado C.D. (2008). Strongyloides hyperinfection: A treatment dilemma. Am. J. Med. Sci..

